# Thread as a Low-Cost Material for Microfluidic Assays on Intact Tumor Slices

**DOI:** 10.3390/mi10070481

**Published:** 2019-07-17

**Authors:** Maxwell Rumaner, Lisa Horowitz, Avital Ovadya, Albert Folch

**Affiliations:** 1 Department of Bioengineering, University of Washington, 3720 15th Ave NE, Seattle, WA 98105, USA; 2 Department of Pathology, University of Washington, 1959 NE Pacific St, Seattle, WA 98195, USA

**Keywords:** thread, low-cost assay, microfluidic devices, cancer, chemosensitivity

## Abstract

In this paper we describe the use of thread as a low-cost material for a microfluidic chemosensitivity assay that uses intact tumor tissue ex vivo. Today, the need for new and effective cancer treatments is greater than ever, but unfortunately, the cost of developing new chemotherapy drugs has never been higher. Implementation of low-cost microfluidic techniques into drug screening devices could potentially mitigate some of the immense cost of drug development. Thread is an ideal material for use in drug screening as it is inexpensive, widely available, and can transport liquid without external pumping hardware, i.e., via capillary action. We have developed an inexpensive microfluidic delivery prototype that uses silk threads to selectively deliver fluids onto subregions of living xenograft tumor slices. Our device can be fabricated completely for less than $0.25 in materials and requires no external equipment to operate. We found that by varying thread materials, we could optimize device characteristics, such as flow rate; we specifically explored the behavior of silk, nylon, cotton, and polyester. The incremental cost of our device is insignificant compared to the tissue culture supplies. The use of thread as a microfluidic material has the potential to produce inexpensive, accessible, and user-friendly devices for drug testing that are especially suited for low-resource settings.

## 1. Introduction

Every year, an estimated 8.2 million people die from cancer around the world [1]. Cancer has become one of the leading causes of death globally and over two thirds of those deaths occur in low- and middle-income countries [1,2]. A significant contributing factor in developing countries is the insufficient funding spent on healthcare annually. In Africa, the continental total health expenditure is just $82 per capita [3]. According to the World Health Organization, this spending is sufficient for basic primary healthcare but is unable to fund more advanced health services, such as oncology. There is a clear need for inexpensive medical tools and devices in developing countries.

Another contributing factor to the rise in cancer-related deaths is the difficult and often ineffective treatment that results from the wide variability in cancer types. Unfortunately, even for the same cancer type, individual cancers vary tremendously from patient to patient which often results in one patient responding quite differently from another patient to the same drug. The variability in patient response reduces the treatment success rate of chemotherapy drugs, decreases survival rates, and costs healthcare systems millions in ineffective treatments. An inexpensive method to test patient’s tumors directly for drug sensitivity would help to treat cancer more efficiently and thus more effectively, especially in the third world.

Another gap in cancer treatment is the excessive cost and difficulty associated with developing new chemotherapy drugs. The cost of developing new cancer drugs is skyrocketing, with the median cost of developing a new cancer drug is now about $700 million [4]. Furthermore, 95% of developing drugs do not even make it out of the preclinical stage, largely because animal models do not accurately predict the behavior of drugs in humans [5]. A solution to compensate for the flaws in these animal models would be to study a drug’s behavior directly on human tissue ex-vivo. However, there is a lack of reliable and inexpensive tools to directly perform functional assays on live human tissue [6]. These assays are presently performed mostly in first-world laboratories, where human tissue is difficult to obtain due to strict regulatory approvals. On the other hand, third-world laboratories are subject to much less stringent regulations for access to human tissue, so a low-cost technology that facilitates functional testing would have a deep impact in the oncological landscape of these countries.

Over the past decade, four main cancer screening approaches have emerged that attempt to study drug responses in live tumor tissue: (1) Tumor organoids are the 3D culture of patient-derived cancer cells as spheroids. Tumor organoids can recreate cell–cell and cell–matrix 3D interactions that more closely resemble in vivo interactions than standard 2D cultures; they have been used for high-throughput drug screening assays that can be predictive of the patient’s responses [7,8,9,10,11,12,13]. (2) Microdissected tumor tissue in culture involves the mechanical fragmentation of biopsies which can cause local tissue damage, but has recently been shown to preserve some key pathways for immunotherapy [14,15]. (3) Organotypic tissue slice culture consists of tumor tissue slices cultured on a porous membrane. Slice culture has been successful at maintaining the tumor microenvironment, but has exhibited challenges involved with high-throughput testing [16,17]. (4) Implantable needles utilize the patient’s tumor while it is still in the body. This method potentially produces the most complete tumor microenvironment, but at the potential risk of patient safety [18,19].

Microfluidic technology presents a unique opportunity to solve current issues in today’s cancer screening approaches. Specifically, the high throughput nature of microfluidic devices has shown to be quite effective and valuable in cancer drug development [20]. Devices have been created to test dozens of drug combinations over tumor cells in cell culture models [21] and identify key cancer-indicating proteins and biomarkers in small volume blood samples [22,23]. However, few studies have addressed the issue of studying drug responses in live tumor tissue. Our lab has pioneered a microfluidic approach that overcomes the throughput limitations of tumor slices [24]. However, our previous microfluidic device (fabricated by assembling multiple micro-molded layers) is expensive to manufacture so low-resource clinics and laboratories will not be able to afford it. Here we explored the feasibility of a low-cost version of our device based on using textile threads which are placed in contact with live tumor tissue for the delivery of drugs; the device is printed with a relatively inexpensive and accessible type of 3D printer.

Various inexpensive medical microfluidic devices have been built using thread-based microfluidics [25,26,27,28]. In thread-based systems, the microchannels and control systems of traditional microfluidics are substituted by thin threads to transport fluid via simple capillary action, making the devices simple to use [29]. Furthermore, due to the fact that the microchannels exist as threads, no complex manufacturing is required to construct the channels. We have developed a 3D-printed device that uses an array of biocompatible textile threads (e.g., silk) for the multiplexed delivery of drugs by capillary action in gentle physical contact with live xenograft glioma tumor samples. Fabric has three critical properties—biocompatibility, flexibility, and low cost—which make it highly attractive for building a gentle drug delivery interface that is compatible with live tumor tissue, and that could be inexpensively disseminated to clinical laboratories in low-resource settings.

The device outlined in this report uses an array of silk threads to transport fluid onto the surface of human glioma xenograft tissue slices. The current prototype contains only five distinct delivery lines. However, scaling up the technology would enable testing of more drugs at once. The device can be fabricated completely with a fused deposition modeling (FDM) 3D printer. FDM printers are the most user-friendly type of 3D printers and they are universally available, making the device extremely accessible and simple to manufacture even in underdeveloped countries. Each device costs less than $0.25 in materials. This work addresses the urgent need to develop better test assays based on intact human cancer tissue that can more closely mimic tumor physiology and predict clinical outcomes better than 2D cell culture systems and animal models. This approach also opens the possibility of chemotherapy testing of tissue slices in lower-resource settings.

## 2. Materials and Methods

**Fused deposition modeling (FDM) fabrication**. The thread-based microfluidic device was developed using 3D CAD software. The device was then 3D-printed in translucent polylactic acid (PLA) using a fused deposition modeling (FDM) 3D printer (FlashForge Creator Pro, FlashForge Corporation, Jinhua, China). The printer’s layer height was set to 80 μm with a first layer height of 200 μm. The print and travel speeds were set to 50 mm/s and 70 mm/s, respectively. The extruder temperature was set to 190 °C and the build plate was unheated. To assist in print adhesion, masking tape was laid on the build plate. With these settings, we were able to fully fabricate the device reliably and accurately in only 30 min using less than $0.01 of PLA filament. We utilized the same materials and printer settings to fabricate the thread characterization test apparatuses.

**Threads and absorbent pads**. Nylon thread was Nymo’s white nylon beading thread, the polyester thread was Coats and Clark’s white polyester thread, and the cotton thread was Coats and Clark’s mercerized white cotton. Two types of silk thread were used: Superior Thread’s Kimono Silk #373 White Rice (white-dyed silk) and #374 Mikimoto (undyed variation). The undyed thread was used during testing with fluorescent dyes because the white-dyed silk would interfere with fluorescent imaging. The amount of thread required for the device costs less than $0.03 across all thread types. Sterlitech glass fiber membrane filters were used as an absorbent substrate to absorb fluid and ensure continued flow through the threads. Typical use of the device required about one absorptive filter paper, which cost slightly more than $0.20. Overall, the cost of the thread and absorbent pad needed for a single use of the device was less than $0.25.

**Device assembly and use**. Before assembly, small slits in the PLA at the top of each well were created using a razor at the locations indicated on the printed device. The threads were threaded through the holes in the bottom of the output and input wells and then wedged into the slits. This technique effectively secured the threads in the device during transportation and use. Absorbent pads were inserted into the outflow wells.

Once the device was assembled, the bottom of the device was dipped into a small water bath for approximately one minute to wet the threads. The wet threads ensured that the flow rate during the initial phase of operation stayed relatively constant throughout the entire duration of operation. Once wet, the threads were gently lowered on top of an organotypic tumor slice; these slices are cultured on the surface of a porous membrane in Transwell inserts, hence our device has been designed to fit inside a Transwell insert and with circular symmetry. Next, the dyes were loaded into their respective wells of our prototype device (see the Supplementary Materials for Video S1: Dye Loading in the Device, demonstrating dye loading in the device). During operation, the drug wells were refilled as necessary. When an absorbent pad appeared to be getting saturated, an additional absorbent pad was placed on top of the previous pads to increase absorption capacity when needed. After operation, the device was removed by carefully lifting it from the surface of the tissue. The tissue was then processed and stained to evaluate function of device.

**Human** glioblastoma multiforme (**GBM) xenograft slice culture**. U-87 MG cells (ATCC, Manassas, VA, USA) were grown in Dulbecco’s Modified Eagle’s Medium (DMEM)/F12 (Invitrogen, Carlsbad, CA, USA) supplemented with 10% fetal bovine serum and penicillin/streptomycin. Cells were passaged every 3–5 days at ~75% confluency. Mice were handled in accordance with a protocol approved by the University of Washington Animal Care and Use Committee. Male immunodeficient nude mice (Taconic, Foxn1 nu, New York, NY, USA) aged to 4–10 weeks were injected subcutaneously in the flank (~1 million cells in 200 μL of serum and antibiotic free medium). Mice with flank tumors were sacrificed before tumor volume reached 2 cm^3^ (2–4 weeks). After sacrifice of the mouse, tumors were removed, and 250-μm-thick slices were cut with a 5100 mz vibratome (Lafayette Instrument, Lafayette, IN, USA) and cultured on top of PTFE Transwell membranes with 0.4 μm pore size (Millipore, Burlington, MA, USA) in 6-well plates. The slice culture medium underneath (1.1 mL) contained Neurobasal-A medium (Invitrogen, Carlsbad, CA, USA) with 25% heat-inactivated horse serum (Sigma, St. Louis, MO, USA), Glutamax (Invitrogen, Carlsbad, CA, USA), 2× penicillin/streptomycin (Invitrogen, Carlsbad, CA, USA), and growth factors (Epidermal Growth Factor 20 ng/mL and Fibroblast Growth Factor 20 ng/mL, Preprotech or Invitrogen). Medium was changed three times per week.

**Functionality assessment using dyes**. Both fluorescent and food dyes were used to visualize the flow and transport of fluid from drug wells, through threads, and onto the surface of the tissue. Fluorescent dyes used were Hoechst (Invitrogen, Carlsbad, CA, USA, 16 μM) and Sytox Green (Invitrogen, Carlsbad, CA, USA, 0.1 μM). Food dyes used were Allura Red (Aldrich Chemical Company, Inc., 5 μM) and FD&C Blue #1 (Spectrum, Stanford, CT, USA, 1 μM). During operation, the transport of the food-coloring dyes onto the tissue was photographed using a Nikon DSLR camera. The Nikon DSLR camera was also used to record videos of food coloring dye flow through each thread examined. Using videos of fluid flow through thread, distance over time measurements were obtained to calculate flow velocity. Flow rate data was calculated by multiplying flow velocity by the thread’s specific fluid volume capacity per unit length. Fluorescent staining of tissue was imaged using a Nikon Eclipse Ti inverted microscope with epifluorescence illumination.

To remove excess dyes after device operation, the tissue was rinsed with Dulbecco’s Modified Eagle’s Medium (DMEM) FluoroBrite. We applied 1 mL of DMEM FluoroBrite below the Transwell insert and 0.5 mL of DMEM FluoroBrite above the surface of the tissue. This procedure was performed at 5 min intervals four times. If indicated, the tissue was submerged in the Sytox Green fluorescent death stain (diluted 1/50,000 in DMEM FluoroBrite) for 1 h. The tissue was re-rinsed using the same protocol as above and imaged.

## 3. Results

**Figure 1:**
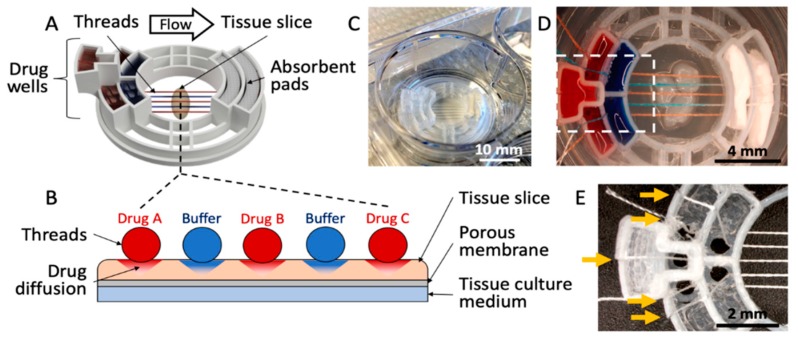
Microfluidic thread device for drug delivery. (**A**) 3D rendering of the device. (**B**) Cross-sectional illustration of the thread-to-tissue fluid transfer concept. (**C**) Photograph of a finished device inside a well of a 6-well culture plate. (**D**) Photograph of the device delivering red and blue dyes to fixed glioblastoma tissue. (**E**) Close-up photograph of an empty device (approximately the area boxed in (**D**), showing the thread-alignment holes at the bottom of the drug wells and the thread-holding slits (yellow arrows) above each well.

As shown in Figure 1, we have developed a 3D-printed microfluidic device that uses threads to deliver fluids to an underlying tissue slice. The device consists of five drug wells, two absorbent pad holders, and a tissue interaction area (Figure 1A). The device transfers fluids from the five drug wells, along the threads that lay across the tissue, then to absorbent pads (Figure 1B). The device measures 2.8 cm in diameter and fits into a standard 3-cm-diameter Transwell membrane insert (used for a 6-well culture plate) (Figure 1C). Up to five threads are evenly spaced across a 4 mm tissue interaction area (Figure 1D). For the prototype shown in Figure 1, the threads are held ~1 mm apart from each other through the use of small holes (1.5 mm in diameter) at the bottom of each well, and slits that hold the strings in place; however, this spacing could in principle be customized. Symmetrical, staggered arrays of these holes on either side of the tissue interaction area ensure the threads are parallel and evenly spaced when in contact with a tissue slice (Figure 1E). The holes do not leak because of their small size and the surface tension of the liquid.

**Figure 2:**
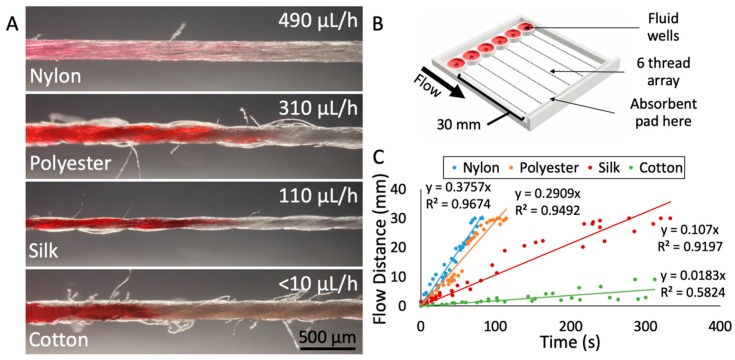
Characterization of different thread materials. (**A**) Photographs of the four threads tested (nylon, polyester, silk, and cotton), with average fluid flow rate indicated. The corresponding flow velocities for each thread are 381 μm/h (nylon), 275 μm/h (polyester), 104 μm/h (silk), and <10 μm/h (cotton). The images were captured at the wicking front with red dye loaded from the left. (**B**) 3D rendering of the test apparatus used to measure flow velocities. (**C**) Results from flow velocity testing of the four thread types. Individual measurements indicated as dots. Five trials were performed per thread type, with six measurements taken per trial.

To determine which thread would be best suited for drug delivery onto tissue, we characterized the physical parameters and the flow-based mass transport of various thread types. For each thread material, we measured the diameter of the thread, the flow rate with an absorbent pad, and the uniformity of the thread’s surface. For our application, the ideal thread should have a small diameter to minimize contact area with tissue, a flow rate that is low enough to maximize run time yet fast enough to prevent drying, and a relatively uniform thread surface (limited number of “side hairs”) to prevent any cross-contamination or unintended fluid transport. We evaluated these key characteristics in four different thread types: Nylon, polyester, silk, and cotton (Figure 2A). Characterization of flow was performed at room temperature, open to the air, with a custom-made 3D-printed device (Figure 2B) with thread holder and wells based on our drug delivery device described above. Red dye solution was applied to prewetted threads and flow distance over time was measured to produce an average flow velocity for each thread type (Figure 2C). One aspect of the device that may have created variation in the flow rate data was the tension in the thread. Juncker and coworkers have observed that the flow resistance of 10 mm of yarns containing a knot depends strongly on the force used to tighten the knot [30]. Similarly, threading the device by hand created variation in thread tension between all threads. Tension on the thread would reduce the fluid capacity per unit length as the cross-sectional diameter and thus the internal space to hold fluid would decrease. In future devices, the thread tension would need to be a controlled factor. To mitigate the effects of the variance in tension in this device, five trials were performed with each of the four threads.

Silk thread proved to be the material best-suited for our application. The diameter of the silk thread was 150 ± 13 μm, with small fluctuations due to the nature of the spun fibers in the thread. The surface of the silk thread was also relatively uniform, with less than one loose thread or “hair” protruding from the surface per millimeter, on average. In contrast, cotton had roughly 10 loose hairs per millimeter and polyester had ~4 hairs per millimeter. Lastly, the flow rate through silk was relatively slow at 110 μL/h. The slow flow rate of dye through silk was ideal because it would not deplete the drug well too rapidly but would be fast enough to sustain flow. Nylon thread exhibited relatively fast flow (490 μL/h) (see the Supplementary Materials for Video S2: Changing from Red Dye to Blue Dye in Device, depicting flow through nylon thread), as did polyester (310 μL/h). Cotton’s flow rate was too slow to sustain flow. In addition, many studies support the biocompatibility of silk thread, finding that silk can be used as a biomaterial in tissue engineering of bone, cartilage, tendon, and ligament tissues [31].

**Figure 3:**
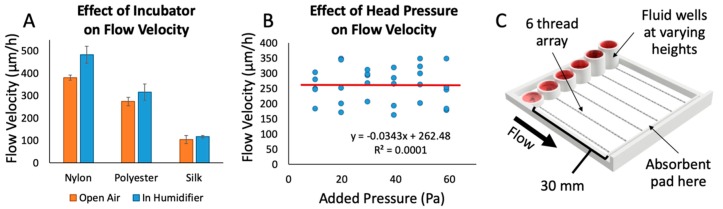
Factors affecting fluid flow. (**A**) The effect of incubator conditions (~97% humidity at 37 °C) on flow velocity through various thread types, performed with red dye and the device shown in Figure 2B. The trend of a 5–20% increase in the average of flow velocities was not statistically significant (Student’s *t*-test, *p*-value of 0.067 (nylon), 0.75 (polyester), and 0.77 (silk)); *n* = 10 trials/strings per condition. (**B**) The effect of added head pressure on flow velocity through various threads. The R^2^ value of 0.0001 in the linear fit indicates that there is no correlation between flow velocity and head pressure up to 60 Pa (ranging from 1–6 mm fluid column height); *n* = 5 runs per pressure value. (**C**) 3D rendering of the test apparatus used to test the effect of different head pressures on flow velocity.

We also examined the effect of ambient humidity on fluid flow through the threads, since cell culture devices must perform effectively in a warm, humid environment (Figure 3A). We measured the flow rate through threads in an incubator using the device shown in Figure 2B. Each thread was suspended with one end connected to a well with red food-coloring dye and the other end touching an absorbent pad. After 10 min in the incubator, the threads were removed, and the flow rates were recorded. We observed a trend towards increased flow rate that was not statistically significant. The cotton thread did not display any appreciable flow in this time period. The flow rate through silk increased by 5%, polyester increased by 9%, and nylon increased by 19%. Thus, silk is also beneficial in that fluid flow is not highly dependent on ambient humidity.

In order to better understand the parameters that could modify flow rate while running an experiment, we investigated what effects the head pressure from the fluid column at the beginning of the thread might have on flow rate (Figure 3B). In a traditional free-flowing channel, increasing the fluid column height causes a proportional increase in flow rate due to an increase in head pressure on the channel. Using a 3D-printed test apparatus with varying fluid column heights (Figure 3C) and silk thread, we found no direct correlation between increases in head pressure on the threads and changes in flow rate (400% increase in well height and 6-fold changes in pressure). Thus, flow rate with this thread, fluid, and device configuration appears to be mainly governed by the thread resistance and the pulling of the fluid by the absorbent pad. Using an absorbent pad with a higher rate of absorption would theoretically increase the flow rate through threads. In other words, flow rate through the threads could potentially be fine-tuned with absorbent pads of different absorption rates. Without an absorbent pad, on the other hand, we have observed that all fluid flows out of the thread at the thread-tissue contact area, drowning the sample in fluid and rendering the assay useless. Although we have never reached that point in our configuration, as the absorbent pad becomes closer and closer to saturation we hypothesize that it will reach a point where it would no longer be able to sustain flow at a quick enough rate to prevent excess fluid from being released over the tissue. To avoid saturation of the absorbent pad, the pad was replaced roughly every hour. With the range of fluid heights used in our experiments, we do not anticipate a significant change in flow rate due to differences in input fluid height between wells, and over time. This result allowed us to design the device with a maximum drug well height in order to maximize the total volume of each well.

**Figure 4:**
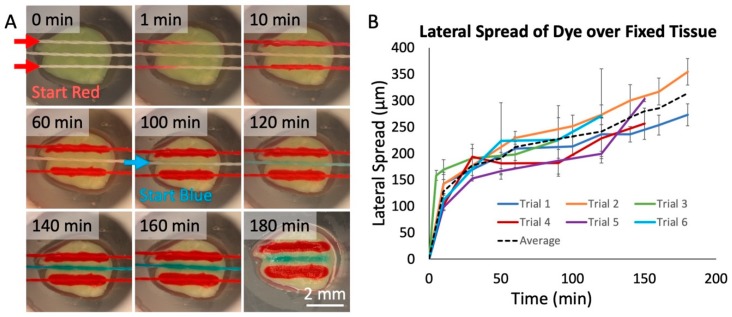
Dye delivery to fixed tissue. (**A**) Red and blue food-coloring dye delivered onto fixed glioblastoma tissue over a 3-hr period in a humidified chamber at room temperature. The two lateral threads flowed red dye. The center thread initially flowed phosphate buffered saline (PBS) buffer, replaced by blue dye at 100 min. After 180 min, the tissue was photographed without the device. (**B**) Measured lateral spread of red dye on fixed tissue over time for six threads in two/three separate device runs. Each measured point was then calculated by taking the average and standard deviation of 10 separate spread measurements at each time, with spread estimated as the total lateral width/2. The black dashed line represents the average of all trials.

To evaluate the transfer of fluid from threads to tissue, we used living and fixed tissue slices from brain xenograft tumors. These brain tumors were generated by injection of cells derived from a patient with glioblastoma multiforme (GBM), the most aggressive and deadly form of brain cancer. We first used food dyes to test whether we could successfully deliver solutes to tissues. As shown in Figure 4A, we selectively delivered dyes to fixed GBM slices with a 3-string device over 3 h. In the first 2 h, two threads delivered red dye, spaced with a thread that delivered phosphate-buffered saline (PBS) buffer in between. During the last hour, the PBS buffer was replaced with blue dye (see the Supplementary Materials for Video S3: Flow Through Nylon Thread, depicting the interchange of red dye to blue dye). The central thread then delivered the blue dye between the two red threads to the tissue. We measured the lateral spread of dye onto the surface of the GBM slices over six distinct trials (Figure 4B). We found a linear increase in spread after the first 10 min of operation, of about 0.8 μm/min. The lateral spread in the first 10 min was significantly quicker (~15 μm/min), likely because of an initial rapid increase in the lateral spread to the width of the silk thread itself (150 μm). To restrict the lateral spread, a “buffer” thread can be used as a sink between two threads to absorb excess spread and prevent cross-talk.

**Figure 5:**
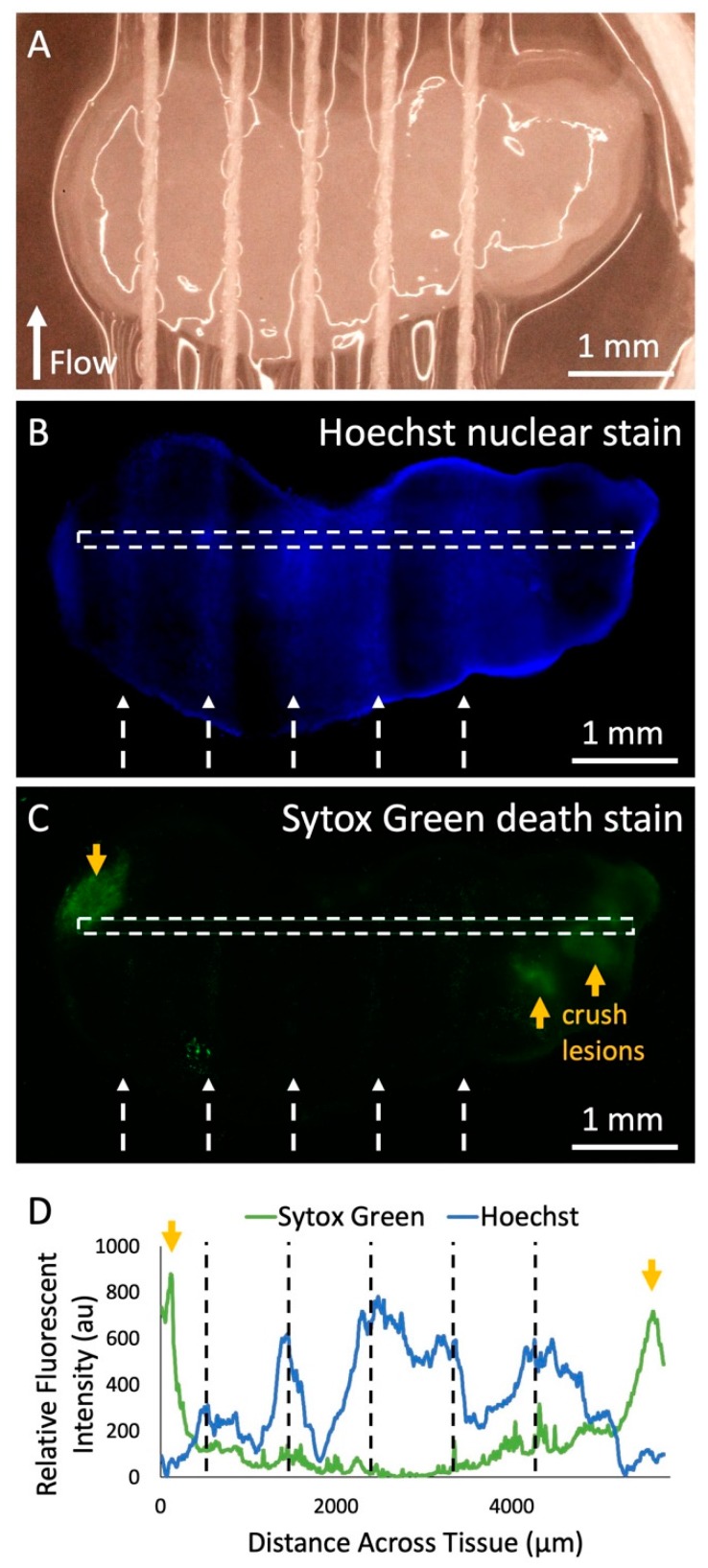
Fluorescent dye delivery to live tissue. (**A**) Photograph of threads across living human U87 glioma xenograft tissue slice in culture before dye delivery with the device. (**B**) Fluorescent image of Hoechst nuclear stain delivery to the tissue after running the device with the Hoechst solution in all lanes for 2 h in the incubator, removal from the device, and staining with the Sytox Green cell death stain. White dashed arrows indicate the thread locations and the direction of flow. (**C**) Fluorescent image of Sytox Green death stain of the tissue. Orange arrows indicate locations of intentional crush lesions that act as positive controls for tissue death. (**D**) Hoechst and Sytox Green fluorescent intensity data across the surface of the tissue (marked by white-dashed rectangles in (**B**) and (**C**). Black-dashed lines indicate the location of threads during the experiment.

Next, we demonstrated solute delivery by the device into living tissue using the Hoechst fluorescent nuclear stain. We ran the Hoechst solution through each of five threads onto a living GBM slice over 3 h (Figure 5A). The areas of tissue in direct contact with thread exhibited increased fluorescence from the Hoechst stain compared to the areas without thread contact (Figure 5B). In Figure 5B there is variability in delivery of the Hoechst stain due to the fact that during this test, thread height was lowered in order to ensure thread contact. The secondary goal of this experiment was to evaluate cell viability when in direct contact with threads. We performed a cell death assay with the Sytox Green dead nuclear stain and found that the threads did not cause any signs of significant unwanted tissue damage (Figure 5C). Intentional crush lesions, created on both ends with forceps outside of the thread contact area, acted as positive death controls. When examining the fluorescent profile of the tissue slice (Figure 5D), it can be seen that locations on the tissue where thread was in contact showed up to six times greater Hoechst fluorescent intensity than areas without thread contact. Future experiments would be necessary to explore the extent of drug delivery under the surface, and the effect of alternating “sink” buffer lanes to restrict lateral dye flow. In addition, areas with thread contact displayed eight times less Sytox Green fluorescence than the crush lesions, which was similar to areas without any thread contact at all, suggesting minimal damage by the threads.

## 4. Discussion and Conclusions

In summary, we have developed a thread-based microfluidic device to inexpensively deliver fluids and solutes to subregions of living human tumor tissue slices, an approach that could be potentially adapted for functional drug testing in poor resource settings. After characterization of thread-based fluid transfer with different materials, we evaluated device performance and functionality with human GBM xenograft slices. Using threads, we have shown that multiple fluids can be transported onto a single slice of living human tumor tissue for functional drug testing applications where tumor tissue is scarce. This device was fabricated cheaply and rapidly—its frame can be 3D-printed with a MakerBot-style 3D-printer—and it requires no power, extra equipment, or expertise for its operation—it is just set atop the sample and flow is powered autonomously by absorbent paper. In contrast, other drug delivery methods or microfluidic devices for drug testing require access to costly and complex fabrication methods, facilities, and equipment as well as specialized operation procedures. The low cost of the technique demonstrated here highlights the potential of thread-based microfluidics as a method for testing the effect of drugs on live tissue that is compatible with low-resource settings. The low-cost advantage of this technique is emphasized when considering that, in a clinical setting, devices that come into contact with the body of a patient cannot be reused. Here, we used thread as a microfluidic component not only for cost reasons, but also because the biocompatibility and flexibility of thread are attractive for building a gentle drug delivery interface that is compatible with live tumor tissue. Thread-based devices could thus contribute to make assessment of potential cancer treatments less cumbersome, less expensive, and more accurate in underprivileged areas.

## Supplementary Materials

The following are available online at https://www.mdpi.com/2072-666X/10/7/481/s1, Video S1: Dye Loading in the Device; Video S2: Changing from Red Dye to Blue Dye in Device; Video S3: Flow Through Nylon Thread.
